# Variations in the origin and course of the suprascapular artery: case report and literature review

**DOI:** 10.1590/1677-5449.008117

**Published:** 2018

**Authors:** Rajani Singh

**Affiliations:** 1 All India Institute of Medical Sciences Rishikesh – AIIMS Rishikesh, Department of Anatomy, Uttrakhand, India.

**Keywords:** suprascapular artery, axillary artery, suprascapular notch, transverse scapular ligament, artéria supraescapular, artéria axilar, incisura supraescapular, ligamento transverso da escápula

## Abstract

The suprascapular artery is normally a branch of the thyrocervical trunk of the subclavian artery. During dissection of the left upper limb of a female cadaver, aged 70 years and fixed in 10% formalin solution, the suprascapular artery was observed aberrantly arising from the first part of the axillary artery. Later, it coursed obliquely behind the clavicle bone and brachial plexus to reach the suprascapular notch, where it was accompanied by the suprascapular nerve. Then, both suprascapular nerve and artery anomalously traversed beneath the transverse scapular ligament. It then irrigated the supraspinatus muscles and took part in the anastomosis around the scapula. On the contralateral side there was no abnormality. Variations in the origin and course of suprascapular artery are of immense value to orthopedic and vascular surgeons, angiographists, and anatomists.

## Resumo

A artéria supraescapular normalmente se apresenta como um ramo do tronco tireocervical da artéria subclávia. Durante a dissecção do membro superior esquerdo de um cadáver do sexo feminino com idade de 70 anos fixado em solução de formol 10%, observou-se que a artéria supraescapular tinha origem aberrante na primeira parte da artéria axilar. Mais adiante, a artéria cursava obliquamente por trás da clavícula e plexo braquial até atingir a incisura supraescapular, onde era acompanhada pelo nervo supraescapular. A partir daí, tanto o nervo quanto a artéria supraescapulares atravessavam de forma anômala por baixo do ligamento transverso da escápula. Neste ponto, irrigavam os músculos supraespinhosos e faziam parte da anastomose em torno da escápula. No lado contralateral, não havia anormalidade. Variações na origem e percurso da artéria supraescapular são de grande valor para cirurgiões ortopédicos e vasculares, angiografistas e anatomistas.

## INTRODUCTION

**Figure 1:**
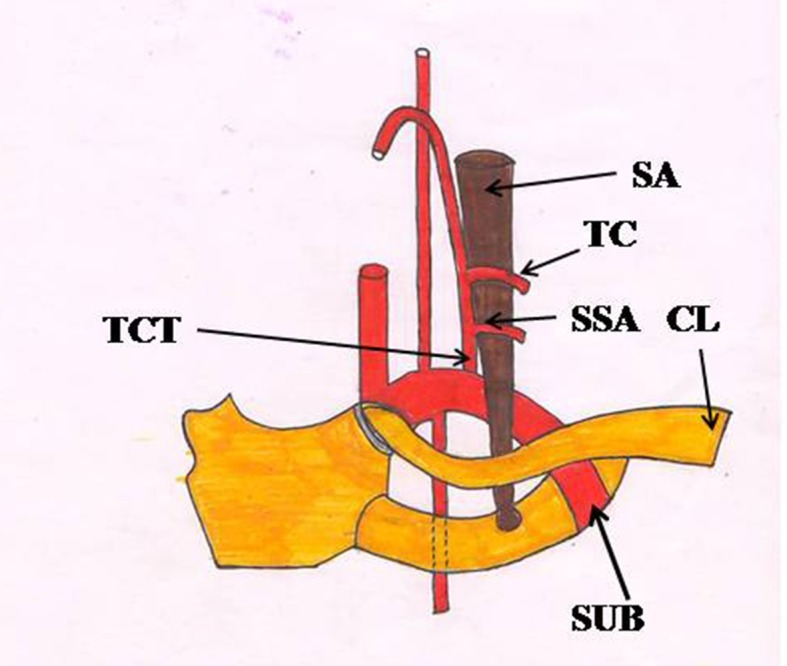
Showing origin of suprascapular artery from the thyrocervical trunk, as described in standard text books. CL, clavicle; SA, Scalenus anterior muscle; SSA, suprascapular artery; SUB, subclavian artery; TC, transverse cervical artery; TCT, thyrocervical trunk.

The suprascapular artery usually originates from the thyrocervical trunk of the subclavian artery (Figure 1), before crossing the laterally situated scalenus anterior muscle, phrenic nerve, brachial plexus, and subclavian artery. On reaching the superior border of the scapula, it crosses over the transverse scapular ligament, while the suprascapular nerve passes beneath the transverse scapular ligament. Numerous variations in the origin and course of the suprascapular artery have been reported by various authors.^1^^,^ 2 One such rare case of anomalous origin and course of the suprascapular artery, arising anomalously from the first part of the axillary artery, is elucidated in the present study. The suprascapular artery irrigates the rotator cuff in general and the supraspinatus muscles, clavicle, acromioclavicular joint, and shoulder joint in particular. Hence, suprascapular artery variants may affect treatments involving the rotator cuff and clavicle fractures. Moreover, during radical and modified neck surgeries, the suprascapular artery must be identified and ligated. If knowledge of variations of the suprascapular artery is absent, mismanagement could occur during these procedures. Furthermore, passage of the artery under the suprascapular foramen could compress neurovascular structures, leading to neurovascular complications. Knowledge of this suprascapular artery variation is also important in cases of arthroscopic suprascapular nerve decompression and in management of glenohumeral region disease due to underlying circulation problems.^2^ Therefore, an origin and course of the suprascapular artery such as that seen in our case takes on great importance. In view of the immense clinical implications of this variation of the suprascapular artery for vascular surgeons, orthopedic surgeons, the case is reported and the related literature has been reviewed.

## CASE REPORT

**Figure 2:**
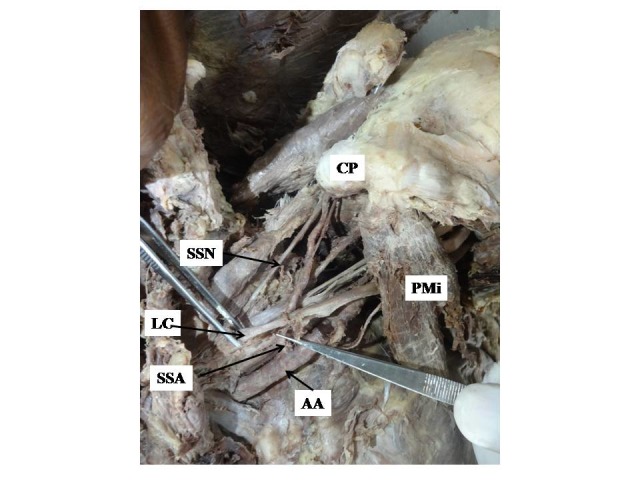
Showing anomalous origin of the suprascapular artery from the first part of the axillary artery. AA, axillary artery; CP, coracoid process; LC, lateral cord; PMi, pectoralis minor; SSA, suprascapular artery; SSN, suprascapular nerve.

During dissection of the left upper limb of a female cadaver, aged 70 years and fixed in 10% formalin, an anomalous origin and course of the suprascapular artery (Figure 2) was observed, differing from what is described in standard text books on Anatomy (Figure 1). A total of 28 upper limbs from 14 cadavers of North Indian origin were observed. Of these limbs, an aberrant origin of the suprascapular artery from the first part of the axillary artery (Figure 2) was detected in only one limb, constituting 3.6% of this sample. It was crossed over anteriorly by the lateral cord of the brachial plexus. Later in its course, it passed obliquely behind the clavicle to reach the suprascapular notch, where it was accompanied by the suprascapular nerve. Both nerve and artery passed beneath the transverse scapular ligament anomalously (Figure 2) and thereafter the artery entered the supraspinous fossa where it irrigated the supraspinatus muscle and took part in the anastomosis around the scapula. There was no abnormality on the contralateral side.

## DISCUSSION

Variant origins and courses of the suprascapular artery have been described earlier by a great number of authors.^1^^,^ 2 Variations in relation to suprascapular vessels and nerves have been classified into four types by Polguj et al.^3^ Our case corresponds to Polguj’s type III. Suprascapular arteries arising from different parts of the subclavian or axillary artery have been observed by Tountas and Bergman^4^ with a median incidence of 10%. Most commonly the artery arises from the subclavian artery, with an incidence of 21.3%.^5^ The suprascapular artery may also originate from the internal thoracic artery (1-5.1%),^5^ from the costocervical trunk (1%),^6^ or from the dorsal scapular artery.^7^ However, in the present study we observed a suprascapular artery that originated from the first part of the axillary artery on the left side in a female cadaver, whereas it typically arose from the thyrocervical trunk on the right side. The origin of suprascapular artery on the left side from the first part of the axillary artery is rare and was reported in one of the cases studied by Mishra and Ajmani.^2^ These authors observed this variation in 1.6% of cases studied. However, the author of the present study observed this variation in 3.8% of cases and crossing of the suprascapular artery by a lateral cord is a rare finding. This discrepancy may be due to different sample size and specimens from a different region. Shukla et al. reported a male cadaver with bilateral suprascapular arteries originating from the first part of the axillary arteries.^8^ A suprascapular artery originating from the first part of the axillary artery on the left side and passing beneath the transverse scapular ligament has been reported in a male cadaver.^9^ Recently, a suprascapular artery arising from the first part of the axillary and piercing the lateral cord was reported.^10^ However, in our case the artery was crossed by the lateral cord of brachial plexus. Naidoo et al. observed this variation in 2% of cases.^11^ Mahato^12^ reported a bilateral variation of the suprascapular artery, where the suprascapular artery arose from the third part of the axillary artery.

An anomalous origin of the suprascapular artery from the third segment of the subclavian artery was observed on the right side in one female Caucasian specimen^13^ (1/62 = 1.6%). However, the suprascapular artery arising from the third part of the subclavian artery has been reported earlier by Reed and Trotter in 25% of cases.^5^ Suprascapular artery emanating from the internal thoracic artery was detected by Reed and Trotter in 10% and in 5% of cases.^5^

Passage of both suprascapular artery and nerve beneath the transverse scapular ligament has been reported by a number of authors.^3^^,^ 4 ^,^ 8 ^,^ 9 ^,^ 12 ^,^ 13 Passage of suprascapular artery beneath the transverse scapular artery was described as subligamentous course by various authors.^5^^,^ 14 According to Tubbs et al.,^15^ the suprascapular artery passed under the superior transverse scapular ligament (STSL) along with the suprascapular nerve with a median incidence of 2.5% (3/120). In one male cadaver, the finding was bilateral and in another male cadaver it was unilateral.^15^

Yang et al.^16^ classified the arrangement of the suprascapular vessels into three types: In Type I (59.4%), all suprascapular vessels passed over the STSL; in Type II (29.7%), the vessels traversed over and under the superior transverse scapular ligament (STSL) simultaneously (at least one vessel passed under or over the STSL); and in Type III (10.9%), all vessels traveled under the STSL. Moreover, three cases of the suprascapular artery passing through the suprascapular notch were detected by Reineck and Krishnan during endoscopic suprascapular nerve release.^17^

A common trunk dividing into the suprascapular and superficial cervical arteries has been described in 30% of individuals, whereas in 28% of the cases both arteries arose directly from the thyrocervical trunk.^4^ There is a report of two cases of duplicated suprascapular arteries existing bilaterally – one arising from the thyrocervical trunk and the other arising from the third part of the subclavian artery. In both cases, the artery with normal origin also had a normal course, whereas the other artery passed below the transverse scapular ligament. The suprascapular artery may be absent in almost 3% of cases.

An origin of the suprascapular artery in the vicinity of the thyrocervical trunk is the most common variation and at the same time it is of least interest from an anatomical or clinical viewpoint, since in this case the course and relations of the artery are not affected.^15^ In contrast, origin of the suprascapular artery from the third part of the subclavian artery or from the axillary artery is rarer, but clinically significant.^13^

Embryologically, all main vessels develop from a primary plexus of smaller ones. During development, some vessels enlarge and form definitive channels and others regress.^7^ During this phase of development, it is possible that different patterns in the vessels may appear, including both the origin and/or the course of either arteries or veins.

## CLINICAL IMPLICATIONS OF VARIANT ORIGIN AND COURSE OF THE SUPRASCAPULAR ARTERY

The suprascapular artery irrigates the tendinous rotator cuff of the shoulder joint. Therefore, variations in the origin of the suprascapular artery will affect management of vascular diseases of the cervical and shoulder regions.^8^

The suprascapular artery irrigates the proximal four-fifths of the clavicle and constitutes the exclusive blood supply for the middle third of the clavicle.^18^^,^ 19 If the suprascapular artery arises from an anomalous position, it is possible that its unusual origin and course could either increase or decrease the chances of being damaged by a fractured clavicle, depending on the proximity of this origin to the middle of the bone. In our case, the suprascapular artery originated from the first segment of the axillary artery and almost behind the external third of the clavicle. In this condition, the probability of damage to artery is lower.

Inside the suprascapular notch, the suprascapular nerve is restrained by the transverse scapular ligament. This may lead to friction, inflammation and finally constriction of the nerve, leading to suprascapular neuropathy.^20^ Ossification of the STSL^21^ and anomalous course of the suprascapular artery under the ligament could lead to constriction of the nerve, since they reduce the capacity of the notch. Chronically, this could lead to atrophy of the supraspinatus and infraspinatus muscles, decreasing abduction and external rotation of the shoulder and causing chronic deep-seated pain in the shoulder that is aggravated with movement. Suprascapular neuropathy may also occur by other mechanisms as well. For example, damage to the suprascapular artery may lead to microemboli in the vasa nervorum of the suprascapular nerve,^3^ and may culminate in ischemia of nerve. Suprascapular nerve injuries have become the main cause of shoulder pain and dysfunction. Hence, knowing the origin and branching pattern of the suprascapular artery would help in the management of diseases of the cervical and shoulder region, which could be of vascular origin.

If a patient presents with shoulder pain which is not due to arthritis, or malfunction of the shoulder's rotator cuff, suprascapular neuropathy must be kept in mind, as the pulsating artery might compress the nerve causing suprascapular neuropathy.^13^

Thus, knowledge of variant origins and courses of the suprascapular artery is invaluable for vascular surgeons, orthopedicians, angiographists, and anatomists.
